# Application-Driven Material Design of Printable Strain Hardening Cementitious Composites (SHCC)

**DOI:** 10.3390/ma15051631

**Published:** 2022-02-22

**Authors:** Egor Ivaniuk, Irina Ivanova, Dmitrii Sokolov, Zlata Tošić, Martin Friedrich Eichenauer, Daniel Lordick, Viktor Mechtcherine

**Affiliations:** 1 Institute of Construction Materials, Technische Universität Dresden, 01062 Dresden, Germany; irina.ivanova@tu-dresden.de (I.I.); dmitrii.sokolov@tu-dresden.de (D.S.); viktor.mechtcherine@tu-dresden.de (V.M.); 2 Research Group Geometric Modeling and Visualization, Technische Universität Dresden, 01062 Dresden, Germany; zlata.tosic@tu-dresden.de (Z.T.); martin.eichenauer@tu-dresden.de (M.F.E.); daniel.lordick@tu-dresden.de (D.L.)

**Keywords:** SHCC, ECC, 3D concrete printing, digital concrete, digital fabrication, additive manufacturing

## Abstract

The creation of concrete shells from customized prefabricated modules is a novel approach that facilitates the construction of free-form surfaces considerably. In the framework of the Adaptive Concrete Diamond Construction (ACDC) project at TU Dresden, a material for 3D printing of the outer contours of such modules has been developed based on the principles of Strain Hardening Cementitious Composite (SHCC). In addition to its high ductility, the required material must also be suitable for 3D printing while enabling the achievement of high geometric accuracy in the manufacture of the modules. To gain the required performance, cellulose ether and starch ether were used specifically to extend the open time, for a longer period of maintaining initial workability, as well as for enhancing shape stability and surface quality. An extensive experimental program was carried out to evaluate the outcomes of the material modifications, including flow table tests, water retention tests, and several specific tests to determine the adhesiveness of the fresh SHCC. For hardened SHCC, surface roughness was assessed using a laser 3D scanner in addition to testing its mechanical properties.

## 1. Introduction

Concrete shells have been known at least since the construction of the Roman Pantheon in the 2nd century AD. Modern thin concrete shells made of reinforced concrete began to appear starting from the early 20th century. The popularity of this design concept increased during the World Wars due to the scarcity of construction materials [1]. Indeed, concrete shells can be by design relatively thin while covering large spans without internal columns or external buttresses, all of which results in significant material savings. According to Gluckovski [2], the use of double-curved shells instead of standard plane slabs reduces the use of material by about 30%. Despite these advantages, concrete shells have rarely been built in recent years. The main reasons for this are the shortcomings of the conventional method for concrete shell construction. This process requires manual erection of complex formwork, see, e.g., [3], which is no longer cost-effective due to an increase in labor costs.

At present, the entire world is faced with the urgent task of minimizing carbon dioxide emissions. The necessary measures include a considerable reduction in the use of building materials, which, in sum, currently account for about 11% of all CO_2_ emissions [4]. Against this background, concrete shells, the construction of which requires relatively small amounts of material, may experience a new dawn. To tackle the problem of inefficiency faced in the conventional method of shell construction, several new methods have been proposed over the years. These include construction using flexible membranes [5,6,7,8], pneumatic formwork [9], pneumatic forming of hardened concrete [10,11], and modularization. Currently, the modularization of concrete shells for later assembly from individual prefabricated modules is the most advanced method, see, e.g., [12], and a significant number of methods for efficient module production, the most widely used of which are adjustable formwork [13] and automated formwork production using CNC-milling [14] or wire-cutting [15].

**Figure 1:**
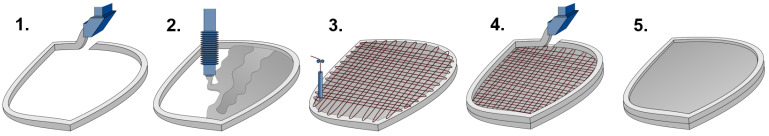
Steps of module production in the ACDC project: steps 1, 4—3D printing of the outer contour, steps 2, 5—infill with SCC by pumping, step 3—the creation of reinforcement mesh.

Being pursued at the TU Dresden Adaptive Concrete Diamond Construction (ACDC) is an ongoing project, the purpose of which is to develop a new approach to the effective and economical production of shell structures from varied, digitally manufactured modules. The project focuses on the design of concrete shells, the creation of algorithms for their segmentation, and the development of technology for automated module fabrication, which consists of several steps according to Figure 1. In the first step, 3D concrete (here: SHCC) printing is applied to produce the outer contour of the module. With this technology, it is possible to create modules with individual shapes without using formwork. As the second step, the area inside the printed contour is filled with self-compacting concrete (SCC) delivered by pumping. Then, the module is reinforced with a mesh made of carbon yarns laid by a robotic arm, an approach similarly suggested in [16]. The flexibility of the carbon yarns enables the creation of reinforcement meshes for modules of any shape, while their high chemical resistance allows for very thin modules with just a few millimeters of concrete cover. Additionally, a specially developed suspension for yarn impregnation increases adhesion to the concrete and improves the reinforcement’s behavior at high temperatures [17]. As the last two steps of module production, the second layer of the outer contour is printed and eventually filled with SCC.

The material used for the 3D printing of the outer edges of the modules must be strong enough to withstand not only quasi-static compressive and tensile forces, including localized loads coming from neighboring modules, but also dynamic forces, which may arise during transportation and assembly. Other requirements for the material are dictated by the need for narrow tolerances in modular construction. Firstly, the outer edges of the modules must be printed with high geometric precision. For this, the print material must provide for the shape stability of the filament during extrusion, and the first layer of the outer edge must not deform during or after deposition of the second layer upon it. Secondly, the material must not be susceptible to shrinkage, which is a particular concern for printed, fine-grain, cement-based materials. Indeed, without a formwork they are not protected from water evaporation during setting and hardening.

Another requirement for the material is the evenness of the printed surface. It must be sufficiently smooth to allow the modules to be directly connected to each other in forming the final structure. Although smooth contact surfaces can be achieved by means of post-processing, the authors’ effort is to avoid methods such as robotic milling or grinding to keep the production chain as efficient and timesaving as possible. Helpful in this context is that it is planned to place elastic rubber gaskets between the modules during assembly. These not only protect the inner zone from precipitation but also make certain tolerances possible in the quality of the contact surfaces by helping to avoid stress concentration spots/zones. Such an approach has already been implemented, for example, in the construction of the Striatus bridge [18].

**Figure 2:**
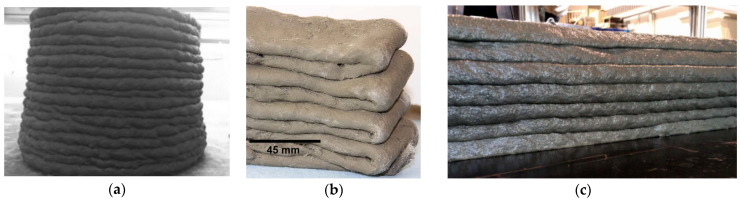
Examples of surface quality of printed SHCC: (**a**) mixture with 2% fiber [25], (**b**) mixture with 2% fiber [28], and (**c**) mixture with 1% fiber [23]; fiber content is given in % by volume.

To date, many types of 3D printing materials have been developed for different applications, varying from lightweight foam concrete [19,20] to ultra-high-performance concrete [21]. From all the available materials, based on its mechanical performance, Strain Hardening Cementitious Composite (SHCC), also known as Engineered Cementitious Composite (ECC), reinforced with short fibers, was chosen as the material for the manufacture of the outer edges of the modules. Due to its fibers, it can withstand relatively high tensile stresses while exhibiting quasi-ductile behavior [22]. SHCC can be produced with low water-to-binder ratios, which enable the attainment of high compressive strength, reaching, if necessary, far beyond 100 MPa [23]. SHCC is also suitable for extrusion, which has been demonstrated in several studies [23,24,25,26]. Li et al. [27] provided a comprehensive review of the progress in the development of printable SHCC. However, the state-of-the-art necessitates further research in developing SHCC suitable for the ACDC project. The main reason for this is that the compositions of printable SHCC developed to date have yielded poor surface quality on the extruded layers; see the examples in Figure 2. Using such material for production of the outer edges of the modules would result in high surface roughness and the consequent inability to connect the modules directly without additional post-processing of the surfaces.

The goal of the present work is to develop a printable SHCC that meets all the requirements for the outer edge material in the ACDC project, including not only adequate mechanical properties, but also parameters that have not been addressed in previously published studies, namely, high shape stability, and acceptable surface quality following extrusion. This publication contains the results of testing the developed compositions in the fresh state, including flow table tests, water retention tests, 3D printing tests, and several specific tests to determine the adhesiveness of the fresh material. For hardened SHCC, surface roughness was assessed using a laser 3D scanner in addition to testing its mechanical properties, which included compression, bending, and uniaxial tension tests.

## 2. Materials and methods

### 2.1. Materials and Mix Design

In this research, a printable SHCC mixture developed by Ogura et al. [23] was chosen as a basis for the reference composition. Such selection was justified by the pronounced strain-hardening behavior of this SHCC under uniaxial tensile loading, while the fiber content, up to 1.5% by volume, in the mixture was lower in comparison to the other SHCC compositions available in the literature, for example, 2% by volume in [24,25,28]. The ultimate tensile strength of the reference SHCC equaled about 5 MPa, and the strain capacity was approximately 3%. While the material exhibited adequate extrudability in its fresh state and satisfactory mechanical performance in its hardened state, the surface quality of the composition was quite poor; see Figure 2c.

To understand the reasons behind this, a reference mixture (Ref) with a composition similar to that presented in [23] was produced and extruded. The reference composition contained no additives other than superplasticizer, which was required to reduce the water content while still demonstrating extrudable consistency. Preliminary printing experiments showed that the poor surface quality of the printed layers could be caused by several factors: Firstly, high fiber content intrinsic to SHCC increases the risk of fiber clumping, which results in the obvious unevenness of the printed surface, such unevenness becoming visually more significant when the material hardens. This problem could be solved partially by adjusting the mixing procedure and by using a 3D printer nozzle with side trowels. Secondly, the reference mixture exhibited troublesome adhesiveness to the tools/equipment. This characteristic reduced the leveling effect of the side trowels due to their surfaces’ dragging on the extruded concrete flowing past. The observed adhesion likely originates from the excessive water content in the very finely grained mixture. The challenge is that the water content cannot simply be reduced, since this would compromise the material’s extrudability. Reducing water content, along with an increase in the dosage of superplasticizer—in order to ensure proper extrudability—is also not a good solution, since the material would, on the one hand, lose its shape stability after deposition and, on the other hand, become even more adhesive due to the higher content of superplasticizer; see [29]. 

Thus, based on the observed behavior of the reference mixture, it was decided to modify it by using chemical additives such as cellulose ether and starch ether. Both these additives are semi-synthetic polymers used in concrete as viscosity-modifying agents [29]. Viscosity-modifying agents are added to concrete to improve its stability, cohesion, and robustness. They are known to increase water retention, thus, also reducing the water loss in the fresh material due to evaporation [29]. This feature made them essential for the dry-mix mortar industry [30]; this feature is promising with respect to its application in printable concretes subjected to surface evaporation of water during the entire period of their setting and hardening. Furthermore, enhanced water retention introduced by use of cellulose ether and starch ether results in prolonged open time, lower shrinkage, and higher crack-resistance of the concrete [31,32,33,34]. Starch ethers are also often used to reduce the adhesiveness in dry-mix mortars [35]. 

In total, three printable SHCC mixtures were developed and compared in this research: (1) the above-mentioned reference composition (Ref), (2) a composition modified with 0.023% of starch ether (SE), and (3) a composition modified using a combination of 0.023% of starch ether and 0.075% of cellulose ether (SE-CE). The dosages are given by the weight of the binder. The binder, comprised of Portland cement CEM I 52.5 R (Holcim Deutschland GmbH, Hamburg, Germany), fly ash Steament^®^ H-4 (Power Minerals GmbH, Germany, Lunen, Germany), and microsilica Emsac^®^ 500E (Master Builders Solutions Deutschland GmbH, Trostberg, Germany), the latter used as a suspension. A higher water-to-binder ratio (w/b) was required for the mixtures with the addition of cellulose ether and starch ether since both substance act as thickeners: w/b was 0.220 for SE and 0.250 for SE-CE in comparison to w/b = 0.212 for Ref. In the case of the reference mixture and SE mixture, the binder composition consisted of 60% by weight of CEM I 52.5 R, 25% wt. of fly ash, and 15% wt. of microsilica (solid substance). In the mixture SE-CE, the content of Portland cement was increased to compensate the possible negative effect of the higher w/b ratio on the mechanical performance of SHCC. Hence, the binder composition in the SE-CE mixture comprised 70% wt. of CEM I 52.5 R, 15% wt. of fly ash and 15% wt. of microsilica (solid substance). Quartz sand fractions 0.06–0.2 mm and 0–1 mm were used as aggregates. The ratio between fractions equaled 30% by volume of 0.06–0.2 mm and 70% by volume of 0–1 mm. The total volume fraction of sand in the concrete was 11.3% and was kept unchanged for all compositions. An amount of 1.5% by volume of polyethylene fiber Dyneema^®^ SK60, with a length of 6 mm, was used in all compositions. Superplasticizer MasterGlenium SKY 593 (Master Builders Solutions Deutschland GmbH, Trostberg, Germany) was employed in all mixtures with the same dosage by weight of binder. Cellulose ether Tylose MH 300 P2 supplied by SE Tylose GmbH & Co., KG (Wiesbaden, Germany) and starch ether Starvis^®^ SE 35 F produced by BASF (Ludwigshafen, Germany) were added to the modified mixtures. All the compositions tested are presented in Table 1.

### 2.2. Characterization of Fresh Material

#### 2.2.1. Flow Table Test

To evaluate the workability of fresh SHCC, flow table tests were performed according to EN 1015-3 [36]. Tests were carried out on three selected compositions within 3 h after water addition and at intervals of 20 min to assess changes in workability over time. To achieve better conformity between the tested material and the material subjected to extrusion in the 3D printing process, the SHCC was remixed for 30 s every 10 min and immediately prior to testing. During each single test, the spread diameter was measured before and after 15 strokes. Additionally, deformations of the material after removal of the steel mold were recorded using a camera installed in front of the flow table.

#### 2.2.2. Water Retention Test

**Figure 3:**
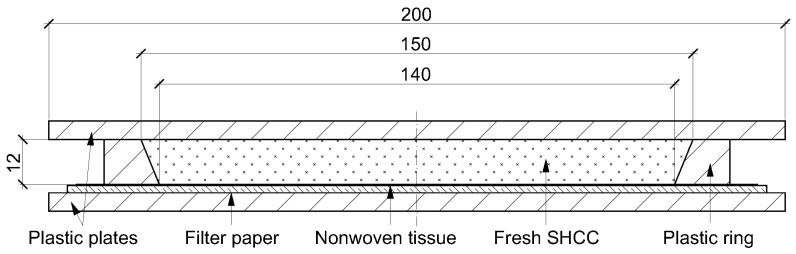
Setup of the water retention test.

Water retention tests were performed according to DIN 18555-7 [37]. The water retention capacity of SHCC was described by the amount of water absorbed by the substrate. The test setup is presented in Figure 3. A thick filter paper covered with a nonwoven tissue was placed on the plastic plate and acted as an absorbent substrate. A plastic conical ring was put on top of the tissue and filled with a portion of freshly mixed SHCC. Subsequently the ring was covered with another plastic plate to prevent moisture evaporation and left for 5 min. The mass of filter paper was measured before and after its contact with the SHCC. The measurements were continued on the same sample for a total of up to 20 min at intervals of 5 min to evaluate the change in water retention of the material over time. Water retention was calculated using Equation (1):

(1) $$WR=100-\frac{W_{s}}{W_{SHCC}}\cdot 100\;\left[\%\right]$$

where $W_{s}$ is the mass of water retained by the substrate and $W_{SHCC}$ is the mass of water in the sample of SHCC placed in the ring.

#### 2.2.3. Evaluation of Adhesiveness

**Figure 4:**
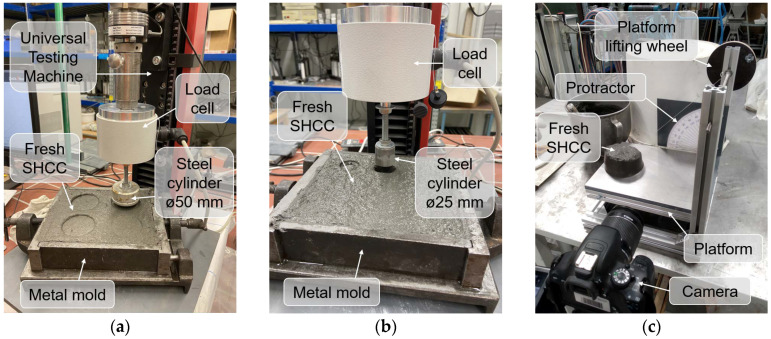
Test setups to assess the adhesiveness of SHCC: (**a**) tack test, (**b**) cylinder pull test, and (**c**) inclined table test.

To assess the adhesiveness of the SHCC mixtures, several tests were performed. The first one, the *tack test* (*TT*), is a typical test used to evaluate the adhesion between hard and soft materials [38]. In the field of 3D concrete printing, this test was used to evaluate the adhesion properties of printable material for rock tunnel lining [39]. In the present investigation, the *TT* was performed as follows: In a metal mold with internal dimensions of 160 mm × 140 mm and a height of 40 mm, SHCC was placed at the top, and the upper surface was leveled. Afterward, a steel cylinder with a diameter of 50 mm was brought into contact and then slightly immersed (6 mm) in the surface of the material inside of the mold with a constant immersion speed of 30 mm/min; see Figure 4a. After a couple of seconds at rest, the cylinder was removed upward at the same constant speed. The forces preventing that movement were caused mainly by the adhesion between SHCC and the bottom surface of the cylinder. Eventually, the adhesiveness of the material was evaluated by the maximum force acting on the cylinder as it moved upward. A total of 8 tests were carried out for each of the tested mixtures.

The second test type, the so-called *cylinder pull test* (*CPT*) shown in Figure 4b is similar to the *TT* but with the differences (a) that the test setup used a steel cylinder with a smaller diameter of 25 mm and (b) was more deeply immersed in the test material, up to 20 mm. SHCC was placed in the same way and in the same steel mold as was done during *TT*. Due to the deeper immersion, the lift of the cylinder was hampered not only by the adhesion forces acting on its bottom surface, but also by the adhesive and frictional forces acting on its sides. Since the frictional stresses were generally low, an increase in the recorded forces as compared to *TT* ensured more prominent differences among the test results for the various materials. In addition, deeper immersion of the cylinder significantly increased the time of the experiment. In order to be able to assign the experiments to a specific age of the material and to compare the results of several successive experiments with each other, the testing time was reduced by increasing the immersion speed to 150 mm/min. For each of the materials investigated, 6 tests were carried out. It is also worth noting that *CPT* can serve a dual purpose: (1) the green strength of the fresh material can be determined when the cylinder moves downwards, i.e., uniaxial confined compression test, and (2) material adhesiveness can be assessed when the cylinder moves upwards.

The *inclined table test* (*ITT*) was used as another method to evaluate SHCC adhesiveness. In this test, the material under examination was put into a mold placed on a horizontal surface that could be tilted as in Figure 4c. To simulate the friction of the material against the steel surface of the 3D printer nozzle trowels and to make the results more comparable with the other two adhesiveness tests, the surface of the setup was covered with a steel plate. A truncated conical mold used in the flow table test was adopted as a mold in *ITT*. After the mold was removed, the slope of the surface with the material placed on it was slowly increased manually. A video-camera was installed in front of the inclined table to record the work. A minimum angle at which the material started to slide across the surface was determined by means of image analysis. It was assumed that a smaller sliding angle was attributable to lower adhesiveness of the material.

#### 2.2.4. 3D Printing Test

**Figure 5:**
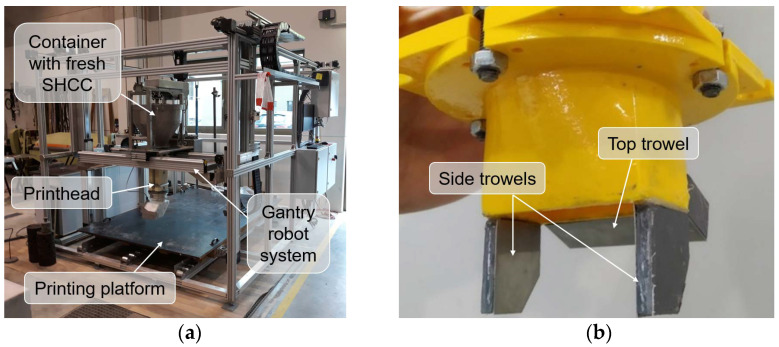
(**a**) Gantry 3D concrete printer and (**b**) vertically oriented nozzle equipped with side and top trowels covered with steel plates.

To verify extrudability, stability of the extruded shape, and surface roughness of the materials under investigation, a gantry 3D concrete printer with a printing area of 1.4 m × 1.0 m and a maximum printing height of 1.2 m was used; see Figure 5a. All the compositions were printed at a constant printing speed of 50 mm/s using a vertically oriented nozzle with outlet dimensions of 55 mm × 25 mm and equipped with side and top trowels covered with thin steel plates; see Figure 5b. The trowels are required to improve the shape and surface quality of the printed filament. To verify the extrudability of the SHCC, specimens of one and two layers were printed. Since the materials were developed for printing, the outer edges of the modules consisting of only two layers for the ACDC project, verification of the buildability of the materials by printing walls with a higher number of layers was not required. After printing all specimens were covered with a polyethylene film and subsequently used to prepare samples for mechanical testing and 3D scanning.

### 2.3. Characterization of Hardened Material

#### 2.3.1. 3D Scanning

**Figure 6:**
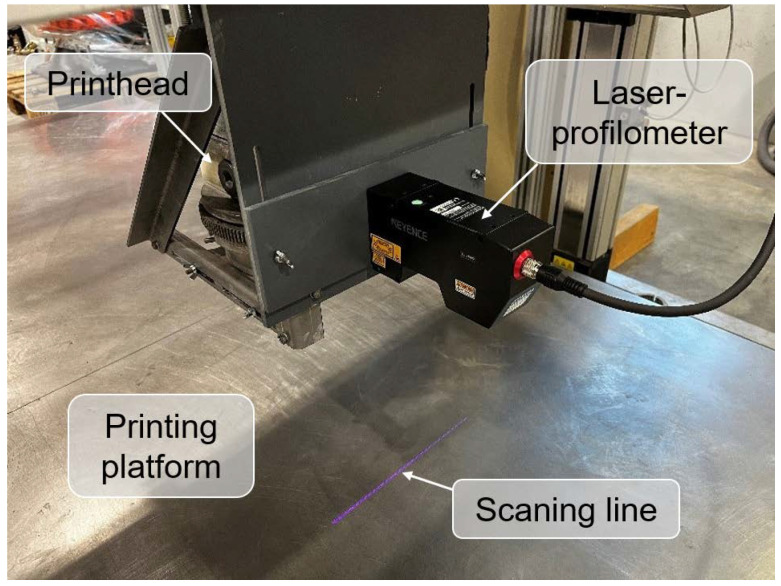
Laser-profilometer Keyence LJ-V7300 mounted on the printhead of the gantry 3D printer and used to evaluate surface roughness.

The assessment of surface quality was carried out using sequential profile scanning, 3D model reconstruction, and roughness calculation. The approach used in this study utilizes the industrial-grade laser-profilometer Keyence LJ-V7300 as shown in Figure 6 to acquire the cross-sectional plane profile of the underlying object, i.e., printed specimens. The scanner sequentially recorded the profiles moving over the scanned area, saving the data for further processing. Five double-layered, printed specimens, each with a length of 40 cm, were scanned for each of the mixtures under investigation.

The reconstruction of the actual shape of the specimens was performed by means of the CON-Print3D User Environment [40], a software package specially developed at TU Dresden for scanning printed objects under flow-through conditions. The software returns a triangulated surface approximation from a point cloud, a 3D model, which is additionally examined concerning surface quality. 

#### 2.3.2. Compression and Bending Tests

**Figure 7:**
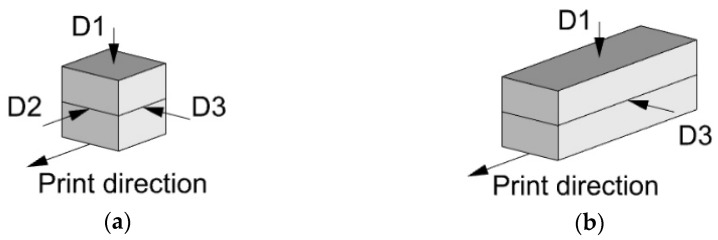
Testing directions in: (**a**) uniaxial compression test and (**b**) bending test.

3D-printed, double-layered specimens were used for production of samples for compression and bending tests. Tests were conducted on the 2nd, 7th, and 28th day after 3D printing. All the samples were cut from the printed specimens, wrapped in a plastic film on the next day after their printing, and stored in a climate chamber at a constant temperature of 20 °C and a relative humidity of 65%. Compression tests were performed on cubes with sides of 40 mm in three different directions, as presented in Figure 7a. For three-point bending tests, rectangular prisms with dimensions of 160 mm × 40 mm × 40 mm were used. The distance between the bottom supports was 120 mm. Prisms were tested in two different directions D1 and D3, as shown in Figure 7b. In all the tests, specimens were loaded with a constant displacement rate of 1 mm/min.

#### 2.3.3. Uniaxial Tension Test

Single-layer 3D-printed specimens were used for the preparation of samples for the tension test. Rectangular prisms with dimensions of 600 mm × 40 mm × 20 mm were cut out from the printed specimens and placed inside a dumbbell-shaped formwork. Then, the empty spaces at both ends of the formwork were filled with fresh SHCC. After curing the fresh material, the samples were removed from the formwork and polished to provide good contact with the grips of the testing machine. Tensile tests were conducted with a constant displacement rate of 1 mm/min. The deformations were measured using Linear Variable Differential Transformers (LVDTs). In addition, a GOM optical measuring system ARAMIS Adjustable 12M [41] was applied to observe crack formation and propagation within the tested specimens.

## 3. Results and Discussion

### 3.1. Fresh Material

#### 3.1.1. Workability

**Figure 8:**
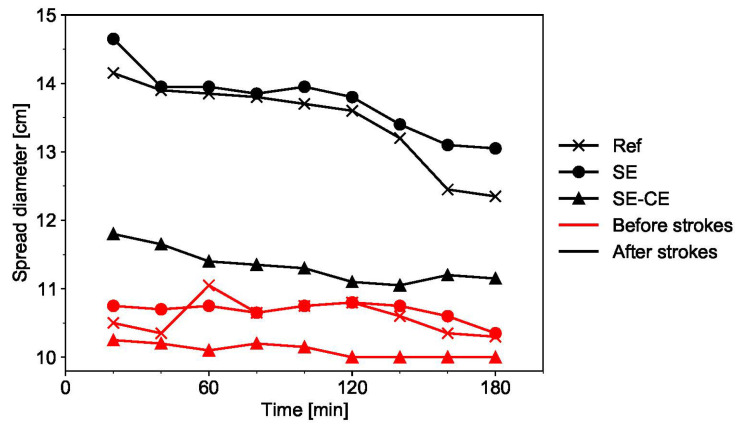
Spread diameter of printable SHCC mixtures over time.

The flow table test results showed that the mixture SE-CE had lower workability in comparison with the mixtures Ref and SE; see Figure 8. This is due to the addition of cellulose ether, which leads to a decrease of the spread diameter of SHCC both before and after the strokes. Moreover, after removing the mold, the mixture SE-CE almost did not spread, i.e., the spread diameter was close to the bottom diameter of the mold. After the strokes, the spread diameter of the same composition SE-CE was also significantly lower than for the two other mixtures.

Concurrently, in comparison with the other compositions, the mixture SE-CE was able to maintain its initial workability for the longest period of time. These results are consistent with the observations of Mueller et al. [42], who showed that cellulose ethers retard the early hydration processes of Portland cement. After three hours, the reductions in spread diameter for mixtures Ref, SE, and SE-CE were equal to 12.7%, 10.9%, and 5.5%, respectively. For Ref and SE, the reduction was already noticeable after two hours, which is undesirable for a printable material since a significant decrease in workability can lead to extrusion problems.

**Figure 9:**
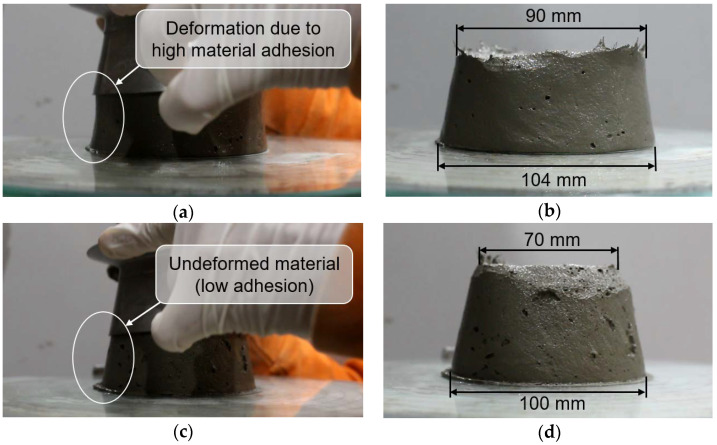
Flow table test; mixture Ref (**a**) during and (**b**) after leveling the form, and mixture SE-CE (**c**) during and (**d**) after leveling the form.

The difference in the adhesiveness of the mixtures studied could be observed in flow table tests during demolding; see Figure 9. For the mixtures Ref and SE in Figure 9a,b, the material adhered to the walls of the mold, thus, forming a more vertically sided sample when the mold was lifted. The SE-CE mixture in Figure 9c,d, in contrast, was able to retain the initial shape of a truncated conical mold.

#### 3.1.2. Water Retention

**Figure 10:**
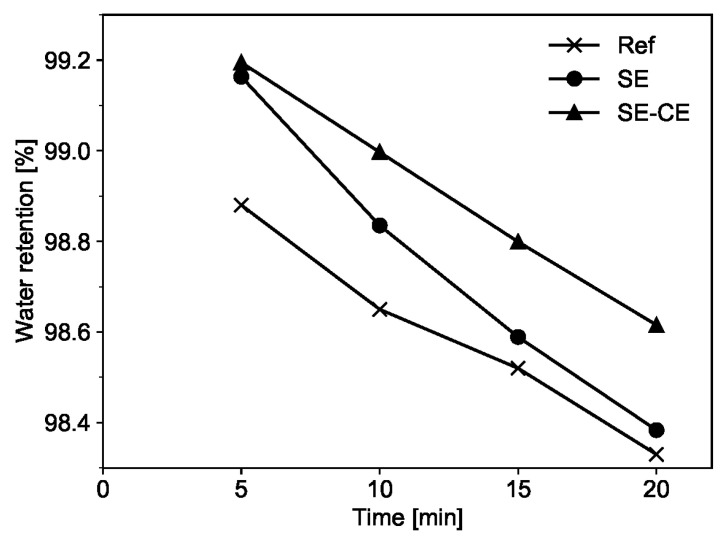
Water retention of printable SHCC mixtures over time.

The results of the water retention test showed that the SE-CE mixture had the highest water retention capacity over time in comparison to the other tested mixtures; see Figure 10. At a material age of 5 min, the mixture SE exhibited a water retention value similar to that of SE-CE; however, its values rapidly reduced over time and, finally, became similar to those for the reference composition at an age of 20 min. 

The high water retention of the SE-CE mixture is attributed to the addition of cellulose ether, which is commonly used as an admixture to improve water retention of cement-based materials [34]. Although starch ether is supposed to provide a similar effect, its efficiency in improving the water retention is lower. High water retention capacity in printable concrete prevents rapid water loss from its surface due to evaporation, to which the material is prone when not protected by formwork.

Based on the results of both the flow table and water retention tests over time, it was decided to proceed with further investigations on the mixtures Ref and SE-CE only and to exclude the composition SE since the SE-CE mixture was characterized by its more highly stable spread flow and its higher water retention over time.

#### 3.1.3. Measurement of Material Adhesiveness

**Figure 11:**
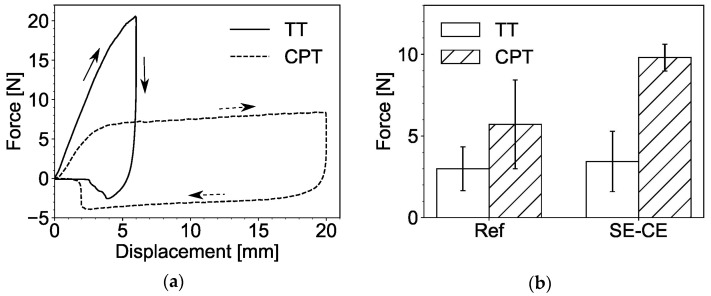
Tack test (*TT*) and cylinder pull test (*CPT*): (**a**) typical force-displacement diagrams, and (**b**) comparison between the results for the mixtures Ref and SE-CE.

Representative force-displacement diagrams for the tack test (*TT*) and the cylinder pull test (*CPT*) are presented in Figure 11a. In these tests, steel cylinders were either brought into contact with the tested material (*TT*) or immersed in it (*CPT*), which yielded positive force values on the graph, and then raised. In both tests, the highest negative values were used to characterize the adhesiveness of the material.

In the *TT*, the cylinder traveled only a short distance, and its lifting was hampered mainly by the force of adhesion between the material and the bottom of the cylinder. The values of this force appeared to be quite low, ranging from 1 N to 7 N, yielding remarkably wide scatter; see Figure 11b. Consequently, *TT* cannot be considered as a sufficiently accurate method to study the adhesiveness of SHCC.

In the *CPT*, the cylinder traveled a greater distance, and its lifting was additionally hindered by adhesive and frictional forces on the sides of the cylinder. Therefore, the measured forces were larger, and their values were more stable from test to test; see Figure 11b. According to the test results, mixture SE-CE yielded a significantly higher value of force and, therefore, must be considered more adhesive than the reference composition. However, this result contradicts the observations made in the flow table tests and during working with both mixtures using trowels and other metal tools as well. A possible reason for this discrepancy is the mixture’s high water retention capacity due to the cellulose ether. In the case of the reference composition, the water promptly appeared on its surface when left at rest. Then, when the steel cylinder came into contact with the sample’s surface; this bleed water served as a lubricant, thus, reducing the friction between the material and the steel cylinder.

**Figure 12:**
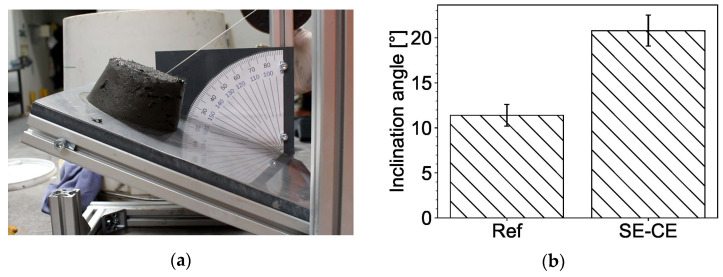
Inclined table test (*ITT*): (**a**) testing process and (**b**) comparison between the results for the compositions under investigation.

The inclined table test (*ITT*) showed good repeatability of the experiments with moderately low scatter, and the tendency observed was similar to that gained from the *CPT*; see Figure 12. As in the case of *CPT*, the possible cause of such result is bleed water appearing at the boundary between the material and the steel surface and acting as a lubricant.

Thus, it may be concluded, that all tests used to assess the adhesiveness of printable concrete, including the tack test, cylinder pull test, and inclined table test, were not able to reflect reality in terms of the comparison between the reference mixture and the SE-CE composition. The reference mixture exhibited higher adhesiveness while working it with metal tools, as well as during flow table tests. However, the proposed tack test, cylinder pull test, and inclined table test led to the result that the mixture SE-CE was more adhesive than Ref. This is likely due to the effect of bleed water, which appeared when the reference material was left at rest and acted as a lubricant between the tested sample and the steel surfaces. To overcome this issue, methods for testing the adhesiveness of the material should be developed that do not take long to prepare, thus, obviating bleeding’s effect on the results. One possible way to assess the material adhesiveness is to observe the shape of the material during the flow table test after lifting the mold, as presented in Section 3.1.1. It is also possible to employ rheometry for the assessment of adhesiveness; research in this area is already underway [43].

#### 3.1.4. 3D Printing Test

**Figure 13:**
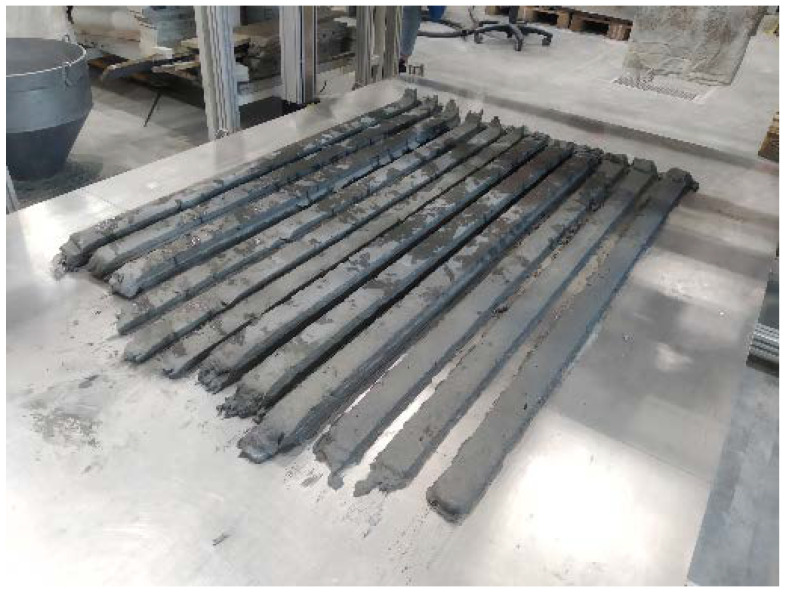
Filaments printed with SHCC mixtures Ref and SE-CE; photo was taken the day after printing and following removal of the cover foil.

The 3D printing test was performed on the SHCC mixtures Ref and SE-CE. Both compositions showed good extrudability; all the filaments were printed without ruptures or significant defects; see Figure 13. No noticeable deformations in the printed layers were found.

### 3.2. Hardened Material

#### 3.2.1. Surface Roughness

The printed specimens were further examined with respect to their surface quality. The 3D models obtained by 3D scanning allowed a comparative analysis of the surface quality among the samples and the addressing of the relationship between surface flatness and the mixture used.

**Figure 14:**
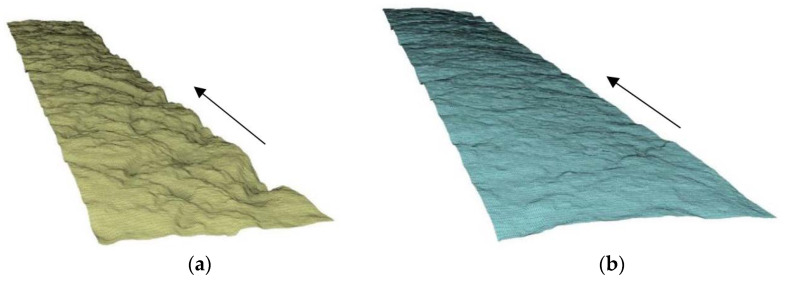
Examples of surfaces with (**a**) high (Ref) and (**b**) low (SE-CE) roughness. Arrows indicate the printing directions.

**Figure 15:**
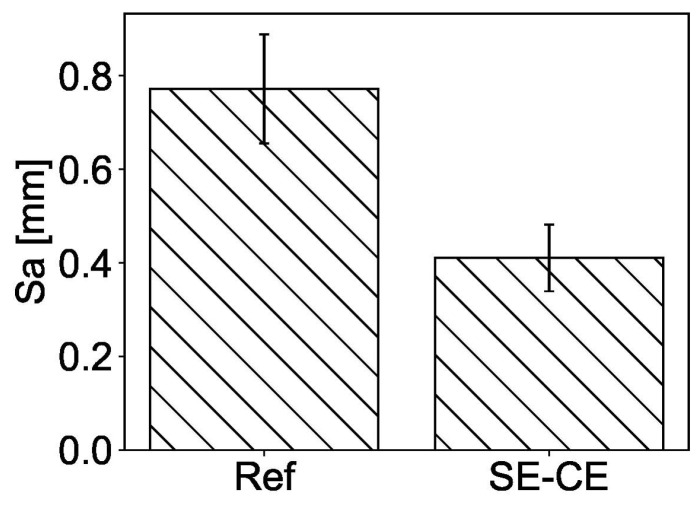
Surface roughness *Sa* for mixtures Ref and SE-CE.

The roughness of the upper surface of the printed specimens was used as a measure of surface quality. The quantification of surface roughness, *Sa*, of the printed layers was performed in accordance with DIN EN ISO 25178 and was based on the calculation of the arithmetical mean deviation of the assessed profile from the reference surface within the sampling area of 50 cm^2^. Figure 14 shows the extreme cases of rough and smooth surfaces of the specimens printed and scanned within the study, corresponding to mixtures Ref and SE-CE, respectively. The scan results are summarized in Figure 15, which shows that the developed composition had a more even surface. For the mixture Ref, the average value of surface roughness Sa was 772 μm, while, for the mixture SE-CE, it was equal to 411 μm.

#### 3.2.2. Ultimate Compressive and Flexural Strength

The mean values obtained for ultimate compressive strength and ultimate flexural strength along with their standard deviations, given in parentheses, are presented in Table 2 and Table 3, respectively.

**Figure 16:**
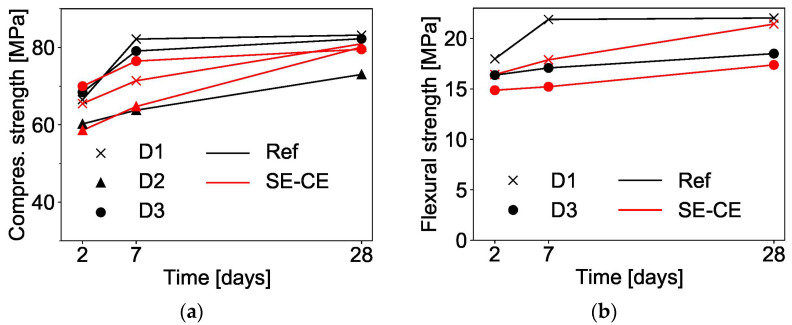
(**a**) Compressive strength and (**b**) flexural strength of printed SHCC.

The SHCC mixtures Ref and SE-CE delivered similar performance in the compression tests; see Figure 16a. For both compositions, the test results in direction D2 were slightly lower than in the directions D1 and D3. This may be due to material anisotropy and the fact of D2’s being parallel to the printing direction, in which the fibers are predominantly oriented [27]. The other two directions D1 and D3 are perpendicular to the printing direction. The results obtained correspond with the observations of Figueiredo et al. [26], who also observed reduced values of compressive strength in the direction of printing.

In the bending tests, the mixture developed using starch ether and cellulose ether (SE-CE) showed slightly lower results than the reference composition; see Figure 16b. The difference between the flexural strength in different directions is also noticeable. For both compositions, bending in the direction D3 showed lower results than in the direction D1. This may be due to the influence of the interlayer bond, in which the material is comparatively weaker.

#### 3.2.3. Ultimate Tensile Strength, Strain Capacity and Cracking

**Figure 17:**
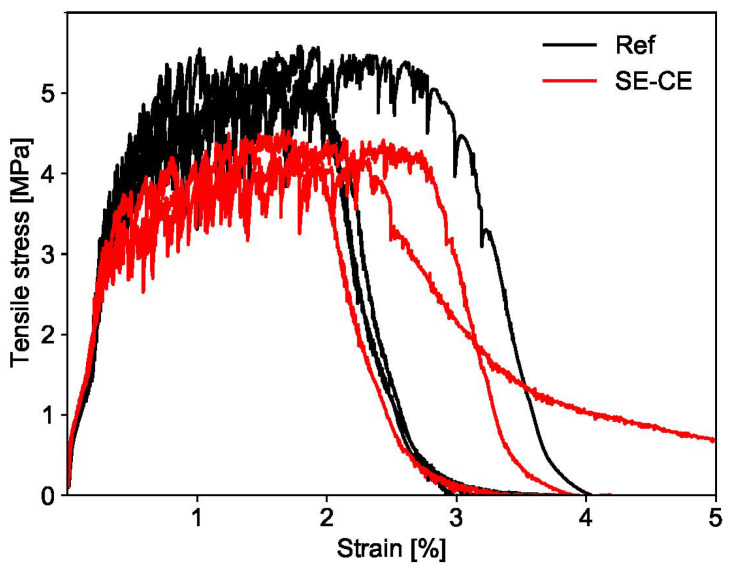
Tensile stress as a function of strain for printed SHCC.

Both mixtures under investigation showed strain hardening behavior under tensile loading. The developed SE-CE mixture had a strain capacity similar to the mixture Ref, which lies between 2 and 3%; see Figure 17. However, in case of mixture Ref, the average ultimate tensile strength was equal to 5.4 MPa, while, for the mixture SE-CE this value was lower by 24%, equaling 4.1 MPa. The reason for this is likely the higher w/b of the latter composition.

**Figure 18:**
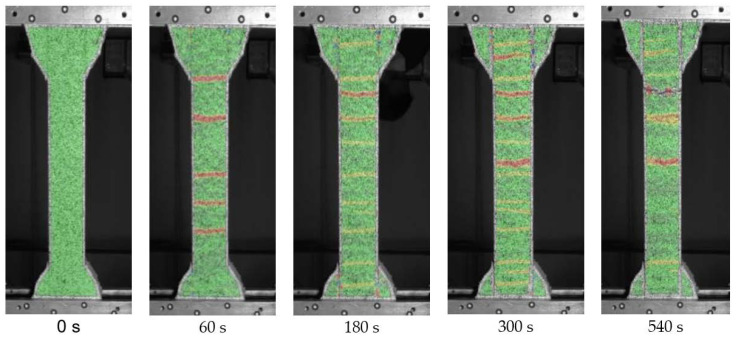
Crack propagation in SHCC samples during uniaxial tension test.

Optical measurements during uniaxial tensile tests showed even distribution of cracks on the surface of the samples; see Figure 18, which may subsequently indicate good distribution of the fibers in the printing direction.

## 4. Conclusions

In the framework of the Adaptive Concrete Diamond Construction (ACDC) project at TU Dresden, a Strain Hardening Cementitious Composite (SHCC) was developed for 3D printing of the outer rims of automatically manufactured modules designed for novel shell structures. The tests conducted in this study showed the following:

- The SHCC, developed with the addition of starch and cellulose ethers, yielded a prolonged period of maintaining initial workability and higher shape stability in comparison to previously developed compositions, as demonstrated in the flow table tests. All these parameters increase the usability of the developed material for 3D printing.
- The developed SE-CE mixture had the highest water retention capacity over time, which is especially important for a 3D printing material, as a high water retention capacity prevents rapid loss of water from its surface.
- Laser 3D scanning of the printed specimens showed enhanced surface evenness of the developed mixture when compared to the reference composition. The smooth surface of the modules printed from the developed material will allow them to be directly connected to each other without additional post-processing.
- The proposed methods for testing the adhesion of the material did not provide adequate results. New methods under development should allow for rapid production of test specimens to avoid bleeding effects.
- The developed SHCC showed high compressive and bending strengths. Considering these two parameters, it is not inferior to the reference mixture. However, due to the increased water-to-binder ratio, which is inevitable in the presence of cellulose ether, mechanical properties were still affected to some extent, i.e., a slight decrease in tensile strength was observed.

Considering all the properties listed above, it can be concluded that the printable SHCC mixture SE-CE as developed can be used for printing the outer contours of modules, from which concrete shells will be assembled. Future studies are planned to investigate the effect of starch and cellulose ethers on the shrinkage of printed SHCC. In this regard a positive influence of such admixtures is expected. 

## Figures and Tables

**Table 1:** Compositions of printable SHCC.

Composition	Ref	SE	SE-CE
Component	Amount per 1 m^3^ [kg]		
Portland cement CEM I 52.5 R	907	895	1013
Fly ash	378	373	217
Microsilica suspension	454	448	434
Sand 0.06–0.2 mm	91	91	91
Sand 0–1 mm	213	213	213
Water	74	84	125
Superplasticizer SKY 593	29.77	29.37	28.50
Polyethylene fiber, L = 6 mm	15.46	15.46	15.46
Starch ether Starvis SE 35 F	-	0.34	0.33
Cellulose ether Tylose MH 300 P2	-	-	1.09

**Table 2:** Ultimate compressive strength of printed SHCC tested in different directions.

Direction	Compressive Strength [MPa]					
	2 Days		7 Days		28 Days	
	Ref	SE-CE	Ref	SE-CE	Ref	SE-CE
D1	66.5 (1.1)	65.5 (3.2)	82.1 (1.4)	71.4 (2.7)	83.2 (6.5)	80.9 (2.4)
D2	60.2 (1.5)	58.6 (1.9)	63.8 (2.8)	64.7 (8.3)	73.0 (11.7)	80.1 (4.7)
D3	68.4 (3.6)	70.0 (4.4)	79.1 (9.8)	76.5 (5.0)	82.2 (11.8)	79.5 (9.3)

**Table 3:** Ultimate flexural strength of printed SHCC tested in different directions.

Direction	Flexural Strength [MPa]					
	2 Days		7 Days		28 Days	
	Ref	SE-CE	Ref	SE-CE	Ref	SE-CE
D1	18.0 (0.6)	16.4 (1.5)	21.9 (1.2)	17.9 (1.6)	22.0 (1.2)	21.4 (1.0)
D3	16.4 (0.8)	14.9 (2.0)	17.1 (0.4)	15.2 (0.5)	18.5 (1.7)	17.4 (1.0)
